# 固相萃取-高效液相色谱法测定水产调味品中7种对羟基苯甲酸酯类防腐剂

**DOI:** 10.3724/SP.J.1123.2022.10004

**Published:** 2023-06-08

**Authors:** Xiaopan NING, Qian YAO, Zhongxiang XU, Yao YIN, Han LIU, Xiaoyan ZHANG, Tao DING, Yong ZHANG, Yu HOU, Mengru WANG, Lina WU, Qiting TANG

**Affiliations:** 1.南京海关动植物与食品检测中心, 江苏 南京 210019; 1. Animal, Plant and Food Inspection Center, Nanjing Customs, Nanjing 210019, China; 2.纳谱分析技术(苏州)有限公司, 江苏 苏州 215123; 2. NanoChrom Technologies (Suzhou) Co., Ltd., Suzhou 215123, China; 3.南京师范大学食品与制药工程学院, 江苏 南京 210097; 3. College of Food and Pharmaceutical Engineering, Nanjing Normal University, Nanjing 210097, China; 4.上海海关动植物与食品检验检疫技术中心, 上海 200002; 4. Animal, Plant and Food Inspection Center, Shanghai Customs, Shanghai 200002, China

**Keywords:** 固相萃取, 高效液相色谱法, 对羟基苯甲酸酯, 蚝油, 虾油, 鱼露, solid-phase extraction (SPE), high performance liquid chromatography (HPLC), parabens, oyster sauce, shrimp sauce, fish sauce

## Abstract

建立了固相萃取-高效液相色谱法同时测定蚝油、虾油、鱼露中的7种对羟基苯甲酸酯类防腐剂(对羟基苯甲酸甲酯、对羟基苯甲酸乙酯、对羟基苯甲酸丙酯、对羟基苯甲酸丁酯、对羟基苯甲酸异丙酯、对羟基苯甲酸异丁酯、对羟基苯甲酸庚酯)。样品分别用甲醇和20%甲醇水溶液超声提取,离心后经MAX固相萃取小柱富集、净化。使用Chromcore 120 C18色谱柱(150 mm×4.6 mm, 3.0 μm)分离,以乙腈-5 mmol/L乙酸铵水溶液为流动相(初始流动相体积比30∶70),梯度洗脱,流速0.7 mL/min,以二极管阵列检测器检测,检测波长254 nm,柱温35 ℃。7种对羟基苯甲酸酯类防腐剂在0.5~50.0 mg/L内具有良好的线性关系,相关系数均大于0.9999,检出限为0.2~0.4 mg/kg,定量限为0.5~1.3 mg/kg。选用蚝油、虾油、鱼露3种样品在3个加标水平下做加标回收试验,7种防腐剂在3种样品中的回收率较好,分别为91.0%~102%、95.5%~106%、95.0%~105%,相对标准偏差≤6.97%。利用该研究建立的检测方法对135个实际样品进行风险筛查,发现某酱油、醋、酱腌菜等存在对羟基苯甲酸甲酯、对羟基苯甲酸乙酯等。该方法操作简单,结果准确,重复性好,干扰少,灵敏度高,可作为测定水产调味品蚝油、虾油、鱼露中7种对羟基苯甲酸酯的有效检测方法。

## Abstract (en)

Seven parabens are widely used in soy sauce, vinegar, jam, oyster sauce, stuffing, and other foods. The long-term intake of large amounts of parabens and similar substances may be harmful to the human body. Therefore, the addition of paraben preservatives to food should be strictly controlled. The current detection method is applicable to single target compound and several food categories, and the experimental pretreatment method involves extraction with anhydrous ethyl ether, which is a toxic reagent. Moreover, interferences in the analysis of parabens via gas chromatography limit the versatility and accuracy of the detection method. Herein, a novel method based on solid-phase extraction (SPE) coupled with high performance liquid chromatography (HPLC) was developed for the determination of seven paraben preservatives (methyl *p*-hydroxybenzoate, ethyl *p*-hydroxybenzoate, propyl *p*-hydroxybenzoate, butyl *p*-hydroxybenzoate, isopropyl *p*-hydroxybenzoate, isobutyl *p*-hydroxybenzoate, and heptyl *p*-hydroxybenzoate) in oyster sauce, shrimp sauce, and fish sauce. Compared with the conventional method, the proposed work enables the determination of more compounds, thereby expanding its scope of application to different food types. This strategy also optimizes the pretreatment method and device parameters. The samples were extracted with methanol and 20% methanol aqueous solution by ultrasonication, respectively, and then centrifuged. The experimental pretreatment method was enriched, and sample clean-up was conducted using a MAX SPE column. The seven parabens were separated using a Chromcore 120 C18 column (150 mm×4.6 mm, 3.0 μm). Gradient elution was performed with acetonitrile-5 mmol/L ammonium acetate aqueous solution as the mobile phase (initial mobile phase volume ratio, 30∶70). The flow rate was 0.7 mL/min, and the column temperature was 35 ℃. A diode array detector with a detection wavelength of 254 nm was also used. The seven paraben preservatives showed good linearity in the range of 0.5-50.0 mg/L, with correlation coefficients greater than 0.9999. The limits of detection (LODs) and quantification (LOQs) for the seven paraben preservatives were 0.2-0.4 mg/kg and 0.5-1.3 mg/kg, respectively. A spiked recovery test was conducted using oyster sauce, shrimp sauce, and fish sauce at three spiked levels of 2, 40, and 200 mg/kg. Good recoveries for the seven paraben preservatives were obtained and the recoveries of the analytes in oyster sauce, shrimp sauce, and fish sauce were 91.0%-102%, 95.5%-106%, and 95.0%-105%, respectively, with relative standard deviations of ≤6.97%. Compared with the liquid-liquid extraction method, the proposed method demonstrated better purification effects. The recoveries of the seven paraben preservatives extracted using this method were also much higher than those obtained from liquid-liquid extraction. We determined the contents of these preservatives in 135 food products using the method established in this study and detected methyl *p*-hydroxybenzoate and ethyl *p*-hydroxybenzoate in soy sauce, vinegar, and pickles. Thus, the established method can be used for the effective determination of seven parabens in aquatic seasoning such as oyster sauce, shrimp sauce, and fish sauce.

对羟基苯甲酸酯又名尼泊金酯,包括对羟基苯甲酸甲酯(MP)、对羟基苯甲酸乙酯(EP)、对羟基苯甲酸丙酯(PP)、对羟基苯甲酸异丙酯(IPP)、对羟基苯甲酸丁酯(BP)、对羟基苯甲酸异丁酯(IBP)、对羟基苯甲酸庚酯(HBP)等,是一种常用的防腐剂,具有低毒性、高效性及良好的抑菌能力^[1]^。我国食品中防腐剂的种类有30多种,常用的防腐剂有山梨酸及其盐类、苯甲酸及其盐类、对羟基苯甲酸酯类等。防腐剂可通过抑制或延迟微生物的生长,防止食品腐败变质,以延长食品保质期。由于山梨酸及其盐类、苯甲酸及其盐类中起主要作用的是分子形式的山梨酸、苯甲酸,需要在酸性条件下才能有效抑制微生物的生长,使苯甲酸、山梨酸的使用范围受到一定的限制。对羟基苯甲酸酯类防腐剂在pH 4~8内能起到很好的抗菌作用^[2,3]^,抑菌范围广,稳定性好,在很多食品中得到良好的应用。对羟基苯甲酸酯类防腐剂的烷基链越长,抑菌效果越好,食品安全性也就更好,其中庚酯较甲酯有更强的抑菌效果,从而可以减少庚酯类的添加量^[4]^,在食品中有良好的应用前景。但对羟基苯甲酸酯类有一定的毒性,大量食用会产生一定的危害,可干扰人体的内分泌系统^[5]^,甚至对人体细胞产生一定的危害^[6]^,如引起过敏性皮炎,对男性的生殖系统产生影响等^[7]^。因此,建立一种准确、高效的检测方法测定食品中的对羟基苯甲酸酯类防腐剂是必要的。

国内外常用的对羟基苯甲酸酯类检测方法有气相色谱法^[8]^、液相色谱法^[9,10]^、薄层色谱法^[11]^、毛细管电泳法^[12]^、高效液相色谱-串联质谱法^[13]^、毛细管胶束电动色谱法^[14,15]^。《食品安全国家标准 食品中对羟基苯甲酸酯类的测定》(GB 5009.31-2016)可测定酱油、醋、饮料及果酱中的MP、EP、PP、BP,检测的目标化合物类别单一,适用食品种类少。气相色谱法的前处理过程使用有毒试剂无水乙醚,且干扰峰对MP和EP的准确积分有影响;薄层色谱法、毛细管电泳法、毛细管胶束电动色谱法的操作简单,但存在分离效果不理想、定量不准确等问题;高效液相色谱-串联质谱法适合痕量分析。这些前处理方法使用单一试剂进行提取,如甲醇、甲醇-水、乙腈-水、正己烷-乙酸乙酯等液液萃取的方式进行提取^[16]^;在这些方法中MP和HBP的回收率相差较大,且均不能同时满足7种化合物的回收率要求。目前尚未有同时测定MP、EP、IPP、PP、IBP、BP、HBP的检测方法。

《食品安全国家标准 食品添加剂使用标准》^[17]^中规定了蚝油、虾油、鱼露、蔬菜、水果、甜面酱、馅料等食品中MP和EP的限量要求。日本、欧盟、美国允许一些常见的对羟基苯甲酸短链酯在食品工业中使用;其中日本规定了果酱、果蔬等食品中PP、IPP、BP、IBP的限量要求^[4]^;欧盟规定了商业产品中每种对羟基苯甲酸酯的最大含量为0.4%,且对羟基苯甲酸酯的总含量不超过0.8%^[18]^;美国规定了发酵麦芽饮料中HBP的限量要求,最高允许限量不超过12 mg/kg^[4]^;香港食物环境卫生署食物安全中心通报某品牌蚝油中违规添加PP^[19]^。因此建立同时测定7种对羟基苯甲酸酯类防腐剂的分析方法就显得尤为重要。

本研究采用固相萃取结合高效液相色谱技术,建立了蚝油、虾油、鱼露中7种对羟基苯甲酸酯类防腐剂的定量分析方法。本工作增加了目标化合物种类,扩充了基质适用范围,优化了前处理方法和仪器参数。本方法选用蚝油、虾油、鱼露等水产调味品做方法学验证,对市售蚝油、虾油、鱼露、甜面酱、果酱、酱油、酱腌菜、醋、果蔬汁饮料等135个实际样品进行了检测,结果表明该方法具有提取效率高、结果准确、重复性好、操作简单、干扰少、灵敏度高、食品种类适应范围广的特点。

## 1 实验部分

### 1.1 仪器、试剂与材料

Brave高效液相色谱分析仪(上海磐诺仪器有限公司); MAX固相萃取小柱(200 mg/6 mL)、ChromCore 120 C18色谱柱(150 mm×4.6 mm, 3.0 μm)、色谱纯甲醇、色谱纯乙腈、色谱纯乙酸铵、0.22 μm有机滤膜(上海安谱实验科技股份有限公司); Lab Dancer涡旋振荡仪(德国IKA公司); SIGMAZ-16K台式冷冻离心机(美国Thermo-Fisher公司); KH-500SP型双频数控超声波清洗器(禾创科技有限公司); SPE469固相萃取装置(北京普立泰科仪器有限公司)。

对羟基苯甲酸甲酯(C_8_H_8_O_3_,CAS号99-76-3,纯度≥99.8%)、对羟基苯甲酸乙酯(C_9_H_10_O_3_,CAS号120-47-8,纯度≥99.7%)、对羟基苯甲酸丙酯(C_10_H_12_O_3_,CAS号94-13-3,纯度≥99.3%)、对羟基苯甲酸丁酯(C_11_H_14_O_3_,CAS号94-26-8,纯度≥99.7%)、对羟基苯甲酸异丙酯(C_10_H_12_O_3_,CAS号4191-73-5,纯度≥99.0%)、对羟基苯甲酸异丁酯(C_11_H_14_O_3_,CAS号4247-02-3,纯度≥99.0%)、对羟基苯甲酸庚酯(C_14_H_20_O_3_,CAS号1085-12-7,纯度≥99.0%)等7种标准品均购买于上海安谱实验科技股份有限公司,且7种标准品均具有标准物质证书。实验中用到的实际样品均为市售样品。

### 1.2 标准溶液的配制

标准储备液的配制:分别准确称取50 mg 7种对羟基苯甲酸酯类标准品,用乙腈溶解并定容至10.0 mL,配制成质量浓度均为5.0 g/L的标准储备液,于4 ℃冷藏保存。

标准中间液的配制:分别准确吸取上述标准储备液2.0 mL,用乙腈定容至100 mL,配制成质量浓度均为100 mg/L的标准中间液,于4 ℃冷藏保存。

混合标准工作液的配制:分别准确吸取上述标准中间液5、10、20、50、100、200、500 μL,并用乙腈定容至1.0 mL,得到质量浓度为0.5、1、2、5、10、20、50 mg/L的7种对羟基苯甲酸酯类化合物混合标准工作液,现配现用。

### 1.3 样品前处理

称取5 g样品(精确至0.01 g)置于50 mL离心管内,涡旋2 min,加入10 mL甲醇,涡旋2 min,超声提取20 min, 8000 r/min离心3 min后取上清液于干净试管中;加入10 mL 20%甲醇水溶液溶解离心后的残渣,涡旋2 min,超声提取20 min, 8000 r/min离心3 min后合并2次提取液,并加水定容至30 mL, 8000 r/min离心3 min。上样前,依次用5 mL甲醇和5 mL超纯水活化MAX固相萃取小柱,取上述离心后的全部上清液作为上样液,经MAX小柱萃取净化;上样后,依次用5 mL超纯水和5 mL 40%甲醇水溶液淋洗,弃去淋洗液,负压抽干2 min;用7 mL甲醇洗脱,收集洗脱液,加超纯水定容至10 mL,过0.22 μm有机滤膜,待高效液相色谱仪分析。

### 1.4 色谱条件

色谱柱:ChromCore 120 C18色谱柱(150 mm×4.6 mm, 3.0 μm);流动相A为乙腈,流动相B为5 mmol/L乙酸铵水溶液;检测器波长:254 nm;流速:0.7 mL/min;进样量:10 μL;柱温:35 ℃;梯度洗脱程序见表1。

**表1:** 梯度洗脱程序

Time/min	φ(A)/%	φ(B)/%	Flow rate/(mL/min)
0	30	70	0.7
8	30	70	0.7
10	40	60	0.7
14	40	60	0.7
19	45	55	0.7
25	45	55	0.7
28	90	10	0.7
32	90	10	0.7
32.1	30	70	0.7
34	30	70	0.7

A: acetonitrile; B: 5 mmol/L ammonium acetate aqueous solution.

## 2 结果与讨论

### 2.1 提取方式的选择

对羟基苯甲酸酯类防腐剂难溶于水,易溶于甲醇、乙腈等有机溶剂^[20]^。实验分别考察了不同提取方式对7种对羟基苯甲酸酯类防腐剂回收率的影响。研究发现,使用单一的甲醇或乙腈作为提取溶剂时,由于7种对羟基链极性、疏水性相差较大,MP和EP的回收率为70%左右;20%甲醇水溶液作为提取溶剂时,HBP的回收率低于85%;使用20%乙腈水溶液提取时,7种对羟基苯甲酸酯的回收率为60%~80%。由此可见,单一使用一种提取溶剂进行提取,不能同时满足7种目标化合物的回收率。使用乙腈和20%乙腈水溶液进行两次提取,基质容易形成分层,普适性低,不易混合过柱;使用甲醇和20%甲醇水溶液进行两次提取,合并提取液,经固相萃取小柱富集、净化,7种对羟基苯甲酸酯类防腐剂的回收率均达到90%以上。因此本方法使用甲醇和20%甲醇水溶液分别提取,可同时满足7种化合物的理化检测要求。7种对羟基苯甲酸酯类混合标准工作液(20 mg/L)的色谱图见图1。

**图1:**
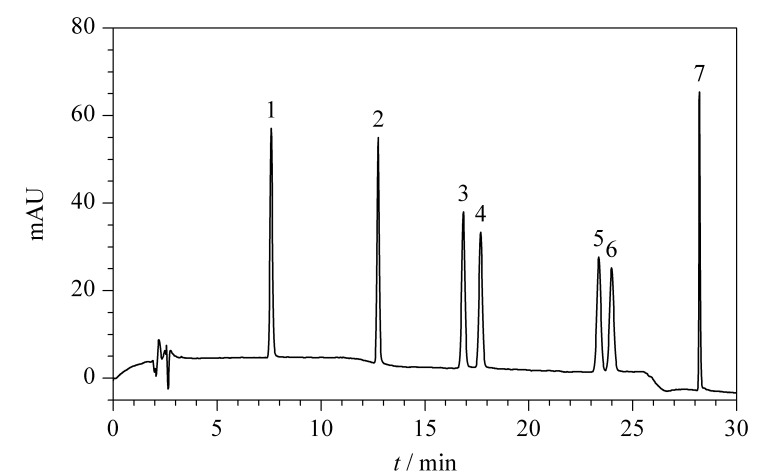
7种对羟基苯甲酸酯类混合标准工作液(20 mg/L)的色谱图

### 2.2 固相萃取小柱的选择

本研究考察了C18、HLB、MAX 3种固相萃取小柱的富集净化效果。使用C18小柱时,7种对羟基苯甲酸酯类化合物的回收率相对较低;与MAX小柱相比,蚝油样品经HLB小柱净化后的颜色更深;样品经HLB小柱与MAX小柱净化后,MP、EP、IPP、PP、IBP、BP等6种化合物的回收率相差不大,但由水产发酵得到的复杂样品,如蚝油样品经HLB小柱净化后,HBP色谱峰出现干扰峰,见图2a,对目标峰的准确积分有影响,不能准确定量;而经MAX小柱净化后的谱图无干扰,见图2b。分析原因可能是小柱之间的净化效果存在差异,经HLB小柱净化后的溶液颜色深,可能会引入更多的杂质,导致HBP色谱峰受到干扰,而经MAX小柱净化后的溶液颜色浅,无干扰。此外,使用MAX小柱净化时,无需调节上样液和洗脱液的pH即可实现7种目标物的洗脱,故最终选择了MAX固相萃取小柱。

**图2:**
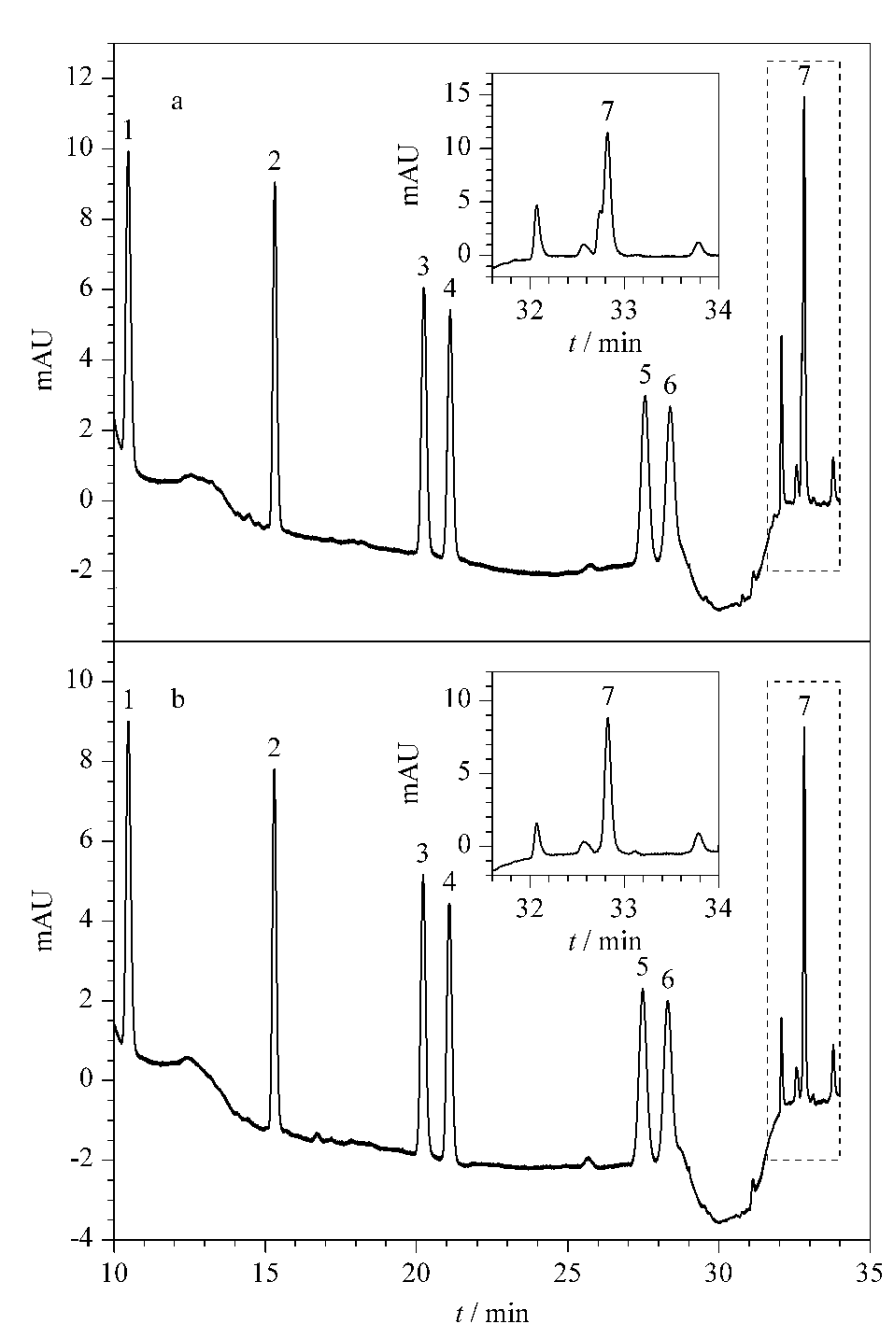
蚝油样品经(a)HLB小柱和(b)MAX小柱净化后的色谱图

### 2.3 流动相的选择

本方法考察了甲醇-水、乙腈-水、乙腈-5 mmol/L乙酸铵水溶液、乙腈-0.1%磷酸水溶液、乙腈-0.1%甲酸水溶液作为流动相时对7种对羟基苯甲酸酯类的分离情况。由于IPP和PP、IBP和BP互为同分异构体,液相色谱中目标峰较难分离,其对流动相梯度的设置与选择较为敏感。甲醇-水作为有机相时,仪器分析时间相对较长且峰形较差;乙腈较甲醇的洗脱能力更强,出峰快,仪器分析时间相对较短。通过优化流动相的比例,7种化合物的目标峰均可以达到很好的分离效果。在水相中加入0.1%甲酸水溶液、0.1%磷酸水溶液、5 mmol/L乙酸铵水溶液作为离子抑制剂,可改善色谱峰拖尾现象。实验结果表明,使用0.1%甲酸水溶液时,进样峰形较宽;使用0.1%磷酸水溶液和5 mmol/L乙酸铵水溶液时的色谱图峰形较好,但磷酸为非挥发性酸,不利于仪器和色谱柱的维护。因此最终确定流动相为乙腈-5 mmol/L乙酸铵水溶液。

### 2.4 检测波长的选择

使用二极管阵列检测器,在190~500 nm波长范围内对7种对羟基苯甲酸酯类化合物的混合标准工作液进行光谱扫描,结果表明检测波长为254 nm和280 nm时响应相对较好。检测波长为280 nm时,IPP、PP、IBP、BP 4种化合物的响应较低;检测波长为254 nm时,7种化合物的响应均很高,灵敏度好。综合考虑7种化合物的灵敏度,选择254 nm作为同时检测7种对羟基苯甲酸酯类防腐剂的最佳检测波长。

### 2.5 方法学验证

#### 2.5.1 线性范围、检出限和定量限

取1.2节混合标准工作液进行检测,以质量浓度(*x*, mg/L)为横坐标、峰面积(*y*)为纵坐标绘制标准曲线,计算回归方程。结果表明,7种对羟基苯甲酸酯类化合物在0.5~50.0 mg/L内线性关系良好,相关系数均大于0.9999,满足标准要求^[21]^。按1.3节方法处理蚝油、虾油、鱼露样品,对得到的样品溶液逐级稀释并检测,以3倍信噪比计算得到的目标组分含量为检出限(LOD),以10倍信噪比计算得到的目标组分含量为定量限(LOQ),数据见表2。

**表2:** 7种对羟基苯甲酸酯类化合物的线性回归方程、相关系数、检出限和定量限

Compound	Linear regression equation	r	Oyster sauce			Shrimp sauce			Fish sauce	
			LOD/ (mg/kg)	LOQ/ (mg/kg)		LOD/ (mg/kg)	LOQ/ (mg/kg)		LOD/ (mg/kg)	LOQ/ (mg/kg)
MP	y=75.31015x+2.38834	0.99999	0.2	0.5		0.3	1.0		0.3	1.0
EP	y=69.87908x+1.45547	0.99994	0.3	1.0		0.4	0.9		0.3	1.0
IPP	y=68.81469x+0.89347	0.99998	0.3	1.0		0.3	1.0		0.4	1.2
PP	y=64.43588x+0.90257	1.0000	0.2	1.0		0.3	1.0		0.3	1.0
IBP	y=62.35856x+1.81123	1.0000	0.4	1.2		0.4	1.3		0.4	1.0
BP	y=61.39980x+1.11312	0.99995	0.3	1.0		0.3	1.0		0.3	1.0
HBP	y=52.83233x+1.16174	0.99993	0.2	0.6		0.3	1.0		0.3	1.0

y: peak area; x: mass concentration, mg/L.

#### 2.5.2 回收率和精密度

选用市售蚝油、虾油、鱼露无本底样品,按1.3节方法处理后分别添加低、中、高3个水平(2、40、200 mg/kg)的对羟基苯甲酸酯类化合物,进行加标回收试验,每个样品做6次平行试验。7种对羟基苯甲酸酯类防腐剂在3种实际样品中的回收率分别为91.0%~102%、95.5%~106%、95.0%~105%,相对标准偏差(RSD)均≤6.97%,结果见表3,所得数据均符合食品理化检测要求^[21]^。

**表3:** 7种对羟基苯甲酸酯类防腐剂在蚝油、虾油、鱼露中的加标回收率(n=6)

Compound	Spiked level/ (mg/kg)	Oyster sauce				Shrimp sauce				Fish sauce		
		Found/ (mg/kg)	Recovery/ %	RSD/ %		Found/ (mg/kg)	Recovery/ %	RSD/ %		Found/ (mg/kg)	Recovery/ %	RSD/ %
MP	2	1.92	96.0	0.425		1.97	98.5	3.27		1.97	98.5	1.90
	40	39.0	97.5	1.59		38.7	96.8	2.35		40.9	102	2.59
	200	205	102	1.63		191	95.5	3.23		194	101	2.58
EP	2	1.82	91.0	0.851		2.03	102	3.71		1.95	97.5	1.39
	40	37.4	93.5	0.997		42.0	105	2.35		41.9	105	1.46
	200	198	99.0	2.05		209	105	3.23		201	101	5.38
IPP	2	1.85	92.5	3.51		2.09	105	2.20		2.06	103	3.04
	40	36.9	92.2	1.16		42.6	106	3.52		41.0	105	2.19
	200	200	100	1.97		208	104	3.37		209	104	3.65
PP	2	1.91	95.5	3.37		2.02	101	5.77		2.07	104	2.62
	40	35.8	93.8	4.24		40.9	102	3.11		40.9	102	2.11
	200	201	100	2.35		207	104	2.56		202	101	1.07
IBP	2	1.90	95.0	2.84		2.05	102	2.22		2.07	104	2.08
	40	37.1	92.8	2.18		41.9	105	3.51		40.5	102	2.09
	200	195	97.5	1.70		206	103	3.70		206	103	2.22
BP	2	1.88	94.0	2.44		2.01	101	3.17		2.08	104	6.97
	40	1.92	96.0	0.425		40.3	101	5.55		40.5	102	4.51
	200	39.0	97.5	1.59		205	102	1.96		200	100	3.30
HBP	2	205	102	1.63		1.91	96.0	2.33		1.90	95.0	3.11
	40	1.82	91.0	0.851		38.8	97.0	0.766		38.8	101	1.58
	200	37.4	93.5	0.997		198	99.0	5.26		193	96.5	2.50

### 2.6 实际样品分析

利用所建立的方法对135个市售样品如蚝油、虾油、鱼露、甜面酱、果酱、酱油、月饼、酱腌菜、醋、果蔬汁饮料等进行测定,并做加标回收试验。

实验结果表明,多个食品中检出对羟基苯甲酸酯类防腐剂,以酱油和醋为例,相应的色谱图见图3,且对羟基苯甲酸酯类防腐剂的回收率较好,数据见表4。其中,MP和EP经常单独或同时应用在酱油和醋中,依据添加剂标准,二者的最大使用限量均为250 mg/kg,虽有添加但符合使用要求;糕点馅料中允许使用此类防腐剂,实验中月饼馅料中MP的检出含量为4.56 mg/kg,可能是由某一种馅料引入的;五香萝卜干和芝麻萝卜干混合使用多种防腐剂以增加防腐效果;混合果汁中检测出EP,含量为2.06 mg/kg,远低于其使用限量,可能由工艺过程或某种果汁代入。以上实验结果进一步证实了该方法能够满足实际样品中对羟基苯甲酸酯类防腐剂的分析。

**图3:**
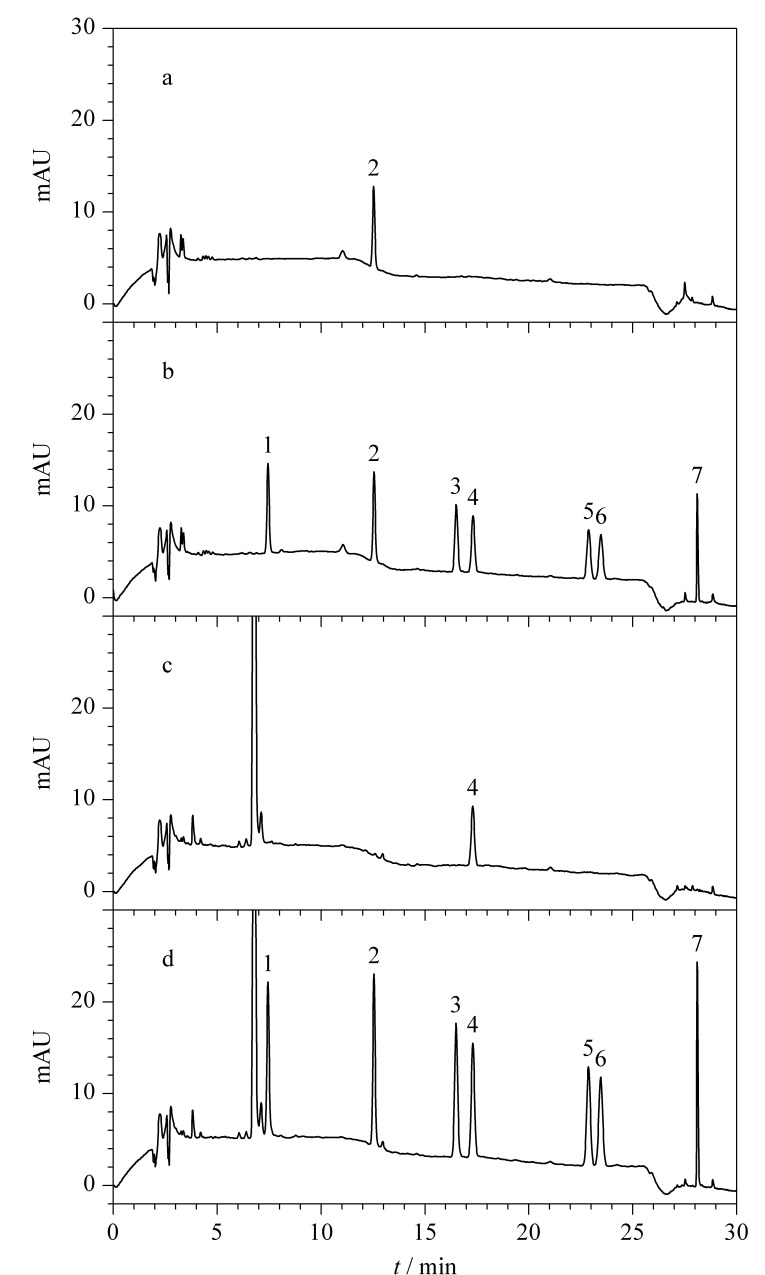
醋和酱油在未加标(a, c)和加标(b, d)条件下的色谱图

**表4:** 实际样品检测结果(*n*=4)

Sample	Compound	Content/ (mg/kg)	Spiked level/ (mg/kg)	Recovery/ %
Soy sauce	EP	50.4	50	98.7
Vinegar	PP	11.2	20	96.0
Moon cake filling	MP	4.56	5	98.1
Mixed juice	EP	2.06	2	97.4
Dried spiced	MP	41.6	50	95.2
Radish	EP	37.7	40	97.0
	PP	28.4	30	95.2
Dried sesame	MP	33.5	30	101.2
Radish	EP	36.3	30	98.7

## 3 结论

本研究建立了固相萃取结合高效液相色谱测定食品中对羟基苯甲酸酯类防腐剂的方法。该法具有提取效率好、回收率高、重复性好、准确度高、适用范围广、操作简单等特点,能较好地满足多种食品中7种对羟基苯甲酸酯类防腐剂的检测需求,同时为进出口食品中防腐剂的监测提供了实用的技术手段。
